# Impact of Combinations of Donor and Recipient Ages and Other Factors on Kidney Graft Outcomes

**DOI:** 10.3389/fimmu.2020.00954

**Published:** 2020-05-22

**Authors:** Maria Gerbase-DeLima, Renato de Marco, Franscisco Monteiro, Hélio Tedesco-Silva, José O. Medina-Pestana, Karina L. Mine

**Affiliations:** ^1^Instituto de Imunogenética, Associação Fundo de Incentivo à Pesquisa, São Paulo, Brazil; ^2^Secretaria do Estado da Saúde, São Paulo, Brazil; ^3^Hospital do Rim, Fundação Oswaldo Ramos, São Paulo, Brazil; ^4^Departamento de Medicina, Universidade Federal de São Paulo, São Paulo, Brazil

**Keywords:** kidney transplantation, donor age, recipient age, death censored graft survival, patient survival

## Abstract

As the availability of kidneys for transplantation continues to be outpaced by its growing demand, there has been an increasing utilization of older deceased donors in the last decades. Considering that definition of factors that influence deceased donor kidney transplant outcomes is important for allocation policies, as well as for individualization of post-transplant care, the purpose of this study was determine the risks for death censored graft survival and for patient survival conferred by older age of the donor in the context of the age of the recipient and of risk factors for graft and/or patient survival. The investigation was conducted in a single-center cohort of 5,359 consecutive first kidney transplants with adult deceased donors performed on non-prioritized adult recipients from January 1, 2002, to December 31, 2017. Death censored graft survival and patient survival were lower in older donors, whereas graft survival was higher and patient survival was lower in old recipients. The analyses of combinations of donor and recipient ages showed that death censored graft survival was lower in younger recipients in transplants from 18 to 59-year old donors, with standard or extended criteria, but no difference in graft survival was observed between younger and older recipients when the donor was ≥ 60-year old. Patient survival was higher in younger recipients in transplants with younger or older donors. Two to six HLA-A,B,DR mismatches, when compared to 0-1 MM, conferred risk for death-censored graft survival only in transplants from younger donors to younger recipients. Pre-transplant diabetes conferred risk for patient survival only in 50–59-year old recipients, irrespectively, of the age of the donor. Time on dialysis ≥ 10 years was a risk factor for patient survival in transplants with all donor-recipient age combinations, except in recipients with ≥ 60 years that received a kidney from an 18–49-year old donor. In conclusion, the results obtained in this study underline the importance of analyzing the impact of the age of the donor taking into consideration different scenarios.

## Introduction

Transplantation is considered the preferred treatment option for patients with end stage renal disease offering survival advantage over long-term dialysis, independently of patient age. As the availability of kidneys for transplantation continues to be outpaced by its growing demand, there has been an increasing utilization of older deceased donors in the last decades (1–6). The proportion of elderly individuals is also increasing among patients on the waitlist (2, 7–9).

With the aim of reducing waiting time for older patients, the Eurotransplant Senior Program or “old for old” was implemented within the Eurotransplant kidney allocation algorithm. This program is based on regional allocation of kidneys from ≥ 65-year old deceased donors to ≥ 65-year old recipients and has been very successful in increasing the number of transplants in elderly recipients (2, 7, 10–12).

The negative impact on kidney graft outcomes of older age of donors and of recipients has been repeatedly reported in the literature (4, 8, 13–15), but there are fewer studies on the impact on graft outcomes of combination of these two variables (16, 17).

Considering that definition of factors that influence deceased donor kidney transplant outcomes is important for allocation policies, as well as for individualization of post-transplant care, the purpose of this study is to investigate the risk for death censored graft survival and patient survival conferred by the combination of the age of the donor and the age of the recipient, along with other factors that may interfere with graft and/or patients survival, such as recipient sex, donor-recipient sex mismatch, pre-transplant diabetes, time on dialysis, cold ischemia time and HLA mismatches (6, 13, 14, 18–38).

## Materials and Methods

### Study Population and Data Source

This is a retrospective single center study on data from 5,359 consecutive first kidney transplants with adult deceased donors performed in non-prioritized adult recipients, from January 1, 2002, to December 31, 2017.

The kidney allocation was performed following the Brazilian national criteria, which is based on HLA-A, B, DR, with emphasis on HLA-DR, compatibility. Kidneys from donors under 18 years of age (not part of this study) are allocated to <18 year-old recipients. In addition, <18 year-old recipients also compete for adult donor kidneys (39). Patients in high risk of losing their last vascular access to dialysis are prioritized on the waitlist and were not included in this study. All the data concerning recipients, donors, and transplant follow-up were obtained from the database of the São Paulo State Registry of Transplants. This registry requests post-transplant follow-up to centers at 3, 6, and 12 months, and yearly thereafter. Failure to comply within 90 days of a request causes a center to have its right to register new patients for transplantation to be suspended until all requested data is provided.

Among the donors, there were 3,066 (57.2%) males and 2,293 (42.8%) females. Four donor age groups were considered: (1) 18–49 years (*N* = 2,783), (2) 50–59 years with standard criteria (SCD) (*N* = 567), (3) 50–59 years with extended criteria (ECD), (*N* = 980), and (4) with 60 or more years (*N* = 1,027). ECD was defined according to the United Network for Organ Sharing, i.e., donors with 60 or more years or with 50–59 years with at least two of these three criteria: history of hypertension, serum creatinine ≥1.5 mg/dL, or death by cerebrovascular accident. For two donors with 50–59 years it was not possible to determine whether they belonged to standard or extended criteria categories and they were excluded from any analysis concerning donor age.

Among the recipients, there were 3,298 (61.5%) males and 2,061 (38.5%) females, 932 (17.4%) had pre-transplant diabetes, and 3,027 (57.1%) were on dialysis for ≥ 10 years. Three age categories were considered: 18–49 years (*N* = 2,730), 50–59 years (*N* = 1,562) and ≥ 60 years (*N* = 1,067).

Cold ischemia time above 24 h occurred in 2,412 (45%) transplants. Concerning HLA compatibility, 1,226 (22.9%) transplants were performed with 0-1 HLA-A,B,DR mismatches.

### Statistical Analysis

The endpoints analyzed were death censored graft survival and patient survival, during the first 5 post-transplant years. Analyses were performed with the GraphPad Prism® 5.0 (GraphPad Software, Inc, La Jolla, CA) and SPSS (Statistical Package for the Social Sciences) (SPSS Inc, Chicago, IL). Graft and patient survival curves were constructed with the Kaplan-Meier method and compared with log rank test or Cox regression analysis. In the Cox regression analyses were included variables with *P*-value < 0.10 in the log rank test. Cases with any missing value were excluded. A two-sided *P*-value ≤ 0.05 was considered statistically significant.

## Results

### Univariate Analyses

The univariate analyses results are presented in Table 1. Donor's older age negatively impacted both death-censored graft (*p* < 0.001) and patient (*p* < 0.001) survival, whereas no impact was observed regarding donor sex. Recipient's older age positively impacted death-censored graft survival (*p* < 0.001) and negatively impacted patient survival (*p* < 0.001). No significant differences were observed regarding recipient sex, although a tendency (*p* = 0.062) was observed toward a higher patient survival in female recipients. Donor-recipient sex mismatch had no influence on death-censored graft or patient survival. Cold ischemia time > 24 h and 2–6 HLA-A,B,DR mismatches impacted negatively on death-censored graft survival (*p* = 0.009 and 0.004, respectively) whereas pre-transplant diabetes and time on dialysis ≥ 10 years had a negative impact on patient survival (*p* < 0.001 for both variables).

**Table 1:** Univariate analysis (log-rank) of the influence of donor, recipient and transplant characteristics on death censored graft survival and patient survival during the first 5 post-transplant years.

**Characteristic**	**Number (%)**	**Missing values, *n***	**5 year death censored graft survival**		**5 year patient survival**	
			**Survival (%)**	***p***	**Survival (%)**	***p***
**Donor age (years)**		2				
18–49	2,783 (52.0)		88.8	<0.001	89.6	<0.001
50–59 SCD	567 (10.6)		88.0		86.0	
50–59 ECD	980 (18.3)		83.7		85.5	
≥ 60	1,027 (19.2)		77.4		84.4	
**Donor sex**		0				
Female	2,293 (42.8)		85.4	0.88	87.2	0.83
Male	3,066 (57.2)		85.9		87.7	
**Recipient age (years)**		0				
18–49	2,730 (50.9)		83.3	<0.001	92.7	<0.001
50–59	1,562 (29.1)		87.3		85.9	
≥ 60	1,067 (19.9)		90.2		76.3	
**Recipient sex**		0				
Female	2,061 (38.5)		86.3	0.53	88.6	0.062
Male	3,298 (61.5)		85.3		86.8	
**Donor-Recipient sex mismatch**		0				
Female-Female	879 (16.4)		85.6	0.64	89.4	0.41
Male-Female	1,182 (22.1)		86.8		88.1	
Male-Male	1,884 (35.2)		85.3	0.86	87.4	0.38
Female-Male	1,414 (26.4)		85.3		85.8	
**Pre-transplant diabetes**		0				
Yes	932 (17.4)		87.3	0.17	80.3	<0.001
No	4,427 (82.6)		85.4		89.0	
**Time on dialysis (years)**		58				
1–9	2,274 (42.9)		83.7	0.28	90.2	<0.001
≥ 10	3,027 (57.1)		86.3		85.5	
**Cold ischemia time (hours)**		2				
0–24	2,945 (55.0)		86.7	0.009	88.1	0.23
> 24	2,412 (45.0)		84.4		86.5	
**HLA-A, -B, -DR mismatches**		0				
0–1 MM	1,226 (22.9)		88.6	0.004	88.7	0.087
2–6 MM	4,133 (77.1)		84.8		87.1	

### Multivariate Analysis

The multivariate analysis included all variables with *p* < 0.10 in the univariate analyses and the results are presented in Table 2. Concerning death censored graft survival, all the variables, except cold ischemia time, remained significantly associated. Regarding patient survival, all the variables with a *p* < 0.05 in the univariate analysis remained significant, whereas sex of the recipient and HLA-A,B,DR mismatches that presented borderline (0.05 > *p* < 0.10) significance in the univariate analysis were not significant in the multivariate analysis.

**Table 2:** Multivariable Cox regression analyses for death censored graft survival and patient survival during the first 5 post-transplant years.

**Variables**	**5 year death censored graft survival**			**5 year patient survival**		
	***p***	**HR**	**95% CI**	***p***	**HR**	**95% CI**
Donor 18–49 years	Reference			Reference		
Donor 50–59 years SCD	0.78	–	–	0.045	1.32	1.01–1.72
Donor 50–59 years ECD	<0.001	1.51	1.23–1.86	0.011	1.32	1.07–1.65
Donor ≥60 years	<0.001	2.11	1.75–2.55	<0.001	1.53	1.25–1.89
Recipient 18–49 years	Reference			Reference		
Recipient 50–59 years	<0.001	0.72	0.60–0.86	<0.001	1.85	1.50–2.27
Recipient ≥60 years	<0.001	0.56	0.44–0.71	<0.001	3.10	2.52–3.82
Recipient sex: male	–	–	–	0.53	–	–
Pre-transplant diabetes	–	–	–	<0.001	1.48	1.22–1.79
Time on dialysis: ≥10 years	–	–	–	<0.001	1.84	1.53–2.21
Cold ischemia time: >24 h	0.082	–	–	–	–	–
HLA-A, -B, -DR: 2–6 mismatches	0.013	1.29	1.06–1.57	0.22	–	–

### Impact of Donor Age on Death-Censored Graft Survival and on Patient Survival

**Figure 1:**
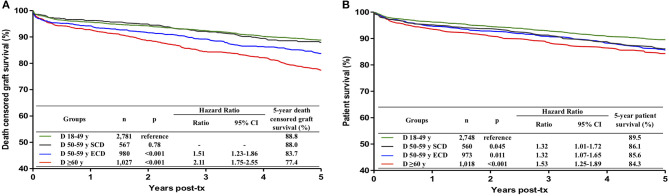
Influence of donor age on death censored graft survival **(A)** and patient survival **(B)** during the first 5 post-transplant years. Donors were divided into four groups, 18–49 years, 50–59 years with standard criteria (SCD), 50–59 years with extended criteria (ECD) and with 60 or more years. ECD were defined according to the United Network for Organ Sharing definition. Kaplan-Meier curves were compared with multivariate Cox regression analysis.

Death-censored graft survival did not differ between 18–49 and 50–59-year old SCD (88.8 vs. 88.0 %, *p* = 0.78). Considering transplants from 18–49-year old donors as reference, graft survival was lower in transplants from 50–59-year old ECD (hazard ratio (HR) 1.51, 95% confidence interval (CI) 1.23–1.86, *p* < 0.001) and from ≥ 60-year old donors (HR 2.11, 95% CI 1.75–2.55, *p* < 0.001) (Figure 1A). The difference in graft survival between 50–59-year old ECD and ≥ 60-year old donors was statistically significant (*p* = 0.002). Considering these results, three age groups of donors (18–59-year old SCD, 50–59-year old ECD and ≥ 60-year old donors) were considered in the remaining analyses.

Patient survival was significantly lower in transplants from donors of any age group > 50 years, as compared to transplants from 18–49-year-old donors (Figure 1B). The patient survival did not differ among transplants from 50–59-year old SCD, 50–59-year old ECD and ≥ 60-year old donors and these three age categories were combined for the remaining analyses.

### Impact of Recipient Age on Death-Censored Graft Survival and on Patient Survival

**Figure 2:**
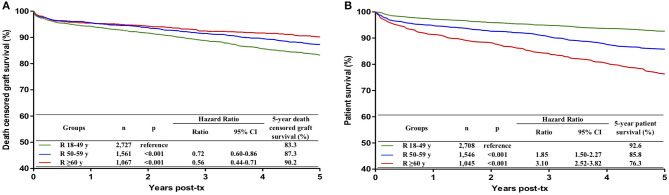
Influence of recipient age on death censored graft survival **(A)** and patient survival **(B)** during the first 5 post-transplant years. Recipients were divided into three groups, 18–49 years, 50–59 years and with 60 or more years. Kaplan-Meier curves were compared with multivariate Cox regression analysis.

Death-censored graft survival was higher in recipient aged 50–59 years (HR 0.72; 95% CI 0.60–0.86; *p* < 0.001) and ≥ 60 years (HR 0.56; 95% CI 0.44–0.71; *p* < 0.001), in comparison with recipients aged 18–49 years (Figure 2A). As the groups with 50–59 years and ≥ 60 years were not significantly different (*p* = 0.093), they were combined for the remaining analyses.

Patient survival was significantly lower in recipients with 50–59 years (HR 1.85; 95% CI 1.50–2.27, *p* < 0.001) and ≥ 60 years (HR 3.10; 95% CI 2.52–3.82, *p* < 0.001), in comparison with recipients aged 18–49 years (Figure 2B). As the groups with 50–59 years and ≥ 60 years were significantly different (*p* < 0.001), the three groups were maintained separately for further analyses.

### Impact of Different Combinations of Donor and Recipient Ages on Death-Censored Graft Survival

**Figure 3:**
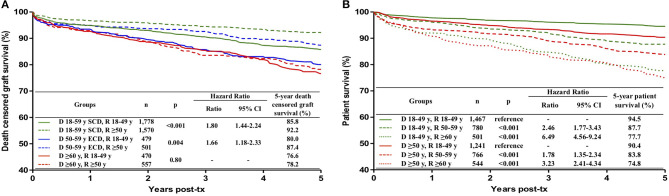
Influence of the combination of donor and recipient ages on death censored graft survival **(A)** and patient survival **(B)** during the first 5 post-transplant years. **(A)** Based on previous results, in death censored graft survival analysis, donors were divided into three groups: 18–59 years with standard criteria (SCD), 50–59 years with extended criteria (ECD) and ≥ 60 years; recipients were divided in two groups: 18–49 years and ≥ 50 years. In patient survival analyses **(B)**, donors were divided into two groups, 18–49 years and ≥ 50 years, and recipients in three groups, 18–49 years, 50–59 years and ≥ 60 years. ECD were defined according to the United Network for Organ Sharing definition. Kaplan-Meier curves were compared with the log rank test.

The results are presented in Figure 3A. Graft survival was lower in 18–49-year old recipients than in ≥50-year old recipients in transplants with 18–59-year old SCD (HR 1.80; 95% CI 1.44–2.24; *p* < 0.001) and with 50–59-year old ECD (HR 1.66; 95% CI 1.18–2.33; *p* = 0.004). There was no difference, however, in the graft survival in younger and older recipients (76.6 vs. 78.2%, *p* = 0.80) when the donor was ≥ 60-year old.

### Impact of Different Combinations of Donor and Recipient Ages on Patient Survival

The results are presented in Figure 3B. In any donor age category, in reference to 18–49-year-old recipients, recipient age of 50–59 conferred a risk for lower patient survival and this risk was even higher in recipients with ≥ 60 years of age. The survival of recipients aged ≥ 60 years did not differ in transplants with 18–49-year old and ≥ 50-year old donors (77.7 vs. 74.8%, *p* = 0.40).

### Impact of HLA Mismatches on Death-Censored Graft Survival in Different Donor-Recipient Ages Combinations

**Figure 4:**
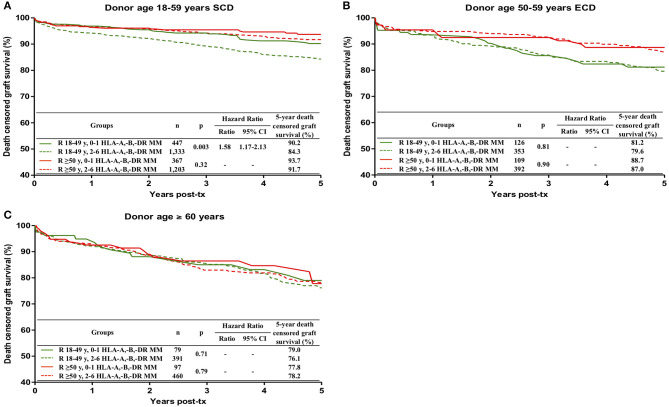
Influence of the combination of recipient age and HLA-A, -B, -DR mismatches on death censored graft survival during the first 5 post-transplant years, stratified by donor age, **(A)** 18–59 years with standard criteria (SCD), **(B)** 50–59 years with extended criteria (ECD) and **(C)** ≥ 60 years. ECD were defined according to the United Network for Organ Sharing definition. Kaplan-Meier curves were compared with the log rank test.

Two to six HLA-A,B,DR mismatches, when compared to 0-1 MM, conferred a significant risk for death-censored graft survival only in transplants from 18–59-year old SCD in 18–49-year-old recipients (84.3 vs. 90.2%, HR 1.58; 95% CI 1.17–2.13; *p* = 0.003) (Figure 4).

### Impact of Pre-transplant Diabetes on Patient Survival in Different Donor-Recipient Ages Combinations

**Figure 5:**
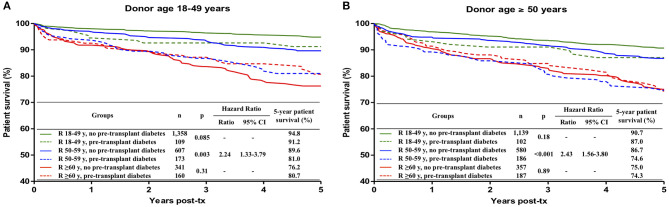
Influence of the combination of recipient age and pre-transplant diabetes on patient survival during the first 5 post-transplant years, stratified by donor age: **(A)** 18-49 years, **(B)** ≥ 50 years. Kaplan-Meier curves were compared with the log rank test.

Pre-transplant diabetes was present in 7.8% of 18–49-year old recipients, in 23.2% of 50–59-year old recipients and in 33.2% of ≥ 60-year old recipients. It was a risk factor for patient survival only in 50–59-year old recipients of kidneys from18–49-year old (HR 2.24; 95% CI 1.33–3.79; *p* = 0.003) and from ≥50-year old (HR 2.43; 95% CI 1.56–3.80; *p* < 0.001) donors (Figure 5).

### Impact of Time on Dialysis on Patient Survival in Different Donor-Recipient Ages Combinations

**Figure 6:**
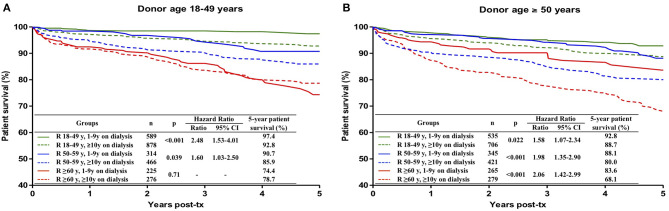
Influence of the combination of recipient age and time on dialysis on patient survival during the first 5 post-transplant years, stratified by donor age: **(A)** 18–49 years, **(B)** ≥ 50 years. Kaplan-Meier curves were compared with the log rank test.

Significantly lower 5-year patient survival in patients with ≥ 10 years on dialysis was observed in transplants with all donor-recipient ages combinations, except in the case of recipients with ≥ 60 years that received a kidney from a 18–49-year old donor. The survival curves and the risk conferred by ≥ 10 years on dialysis in each donor-recipient age combination are presented in Figure 6.

## Discussion

In the current scenario of kidney donor shortage, the use of older donors is unavoidably and thus it is important define/quantify the risks conferred by the advanced donor age that could be useful for allocation matters and for individualization of post-transplant care.

The purpose of this study was to assess the risks for death-censored graft survival and for patient survival conferred by older age of the donor in the context of the age of the recipient and of other possible or well-recognized risk factors for graft and/or patient survival. The investigation was conducted in a single-center cohort of 5,359 consecutive first kidney transplants with adult deceased donors performed on non-prioritized adult recipients from January 1, 2002, to December 31, 2017. The end-points were death-censored graft survival and patient survival in the first 5 years post-transplant.

The univariate analysis showed that donor and recipient age influenced both graft and patient survival, cold ischemia time and HLA-A,B,DR mismatches had an impact on graft survival, and pre-transplant diabetes and time on dialysis influenced patient survival. All these associations, except for cold ischemia time, were confirmed in multivariate analyses.

Female recipients presented a tendency for higher survival (*p* = 0.062) in the univariate analysis, but this association was not significant in the multivariate analysis and thus was not further analyzed. We believe that our data do not allow a definitive conclusion about the influence of the sex of the recipient on patient survival. On the other hand, we did not find any indication for an impact of donor-recipient sex mismatch on transplant outcomes, corroborating the results of other studies (21, 22).

Increased donor age was associated with lower death-censored graft survival and with patient survival, as already described (4, 6, 8, 13–15). In our study, poorer graft survival started to be observed in transplants with 50–59-year old donors with extended criteria donors, while the impact on patient survival was already observed in transplants with 50–59-year old standard criteria donors.

Recipient age ≥ 50 years was associated with higher graft survival and with lower patient survival, confirming the findings of previous publications (16, 17). As it has been reported that younger recipients present a higher rate of rejection episodes (2, 16, 40), the lower graft survival in younger recipients is probably related to a more vigorous immune response, and perhaps also to a higher rate of non-adherence to treatment in this group of patients. On the other hand, the lower patient survival in older recipients is probably explained by the higher age *per se*, increased rate of co-morbidities and higher susceptibility to infections (41, 42).

Considering the opposite effects of recipient age on graft and on patient survival, we also calculated the overall graft survival, i.e., graft failure defined as death of the patient or return to dialysis, in relation to recipient age (data not shown). The results showed that 5-year overall graft survival was not statistically different (*p* = 0.14) between 18–49-year old (77.5%) and 50–59-year old (75.4%) recipients, but was significantly lower (*p* = 0.002) in ≥ 60-year old recipients (69.5%, HR of 1.28) in relation to 50–59-year old recipients.

An interesting observation was that there was no difference in graft survival in younger and older recipients when the donor was ≥ 60-year old, reinforcing the concept that kidneys from old donors should be preferentially allocated to old recipients. In the Eurotransplant Senior Program the ages of donor and the recipient were set at ≥ 65 years (2, 7, 10–12).

Regarding the interplay between donor age, recipient age and HLA incompatibilities, our data showed that 2-6 HLA-A,B,DR mismatches were significantly associated with lower graft survival only in transplants from 18–59-year old donors with standard criteria into younger (18–49-year old) recipients. The 5-year graft survival of 2–6 HLA mismatched transplants from these donors in younger recipients was 84.3%, in contrast with survivals of 90.2%, in 0-1 mismatched grafts in younger recipients, 93.7% in 0–1 mismatched grafts in ≥ 50-year old recipients, and 91.7% in 2–6 mismatched grafts in ≥ 50-year old recipients. The explanation for these results would be the more robust immune response of the younger recipient and the conclusion would be that mismatched grafts should be avoided in younger recipients. This subject deserves further analyses, not only to confirm these results but also to investigate which kind of HLA mismatch should be considered. For instance, would avoiding HLA-DR mismatches be sufficient?

Pre-transplant diabetes conferred a significant risk for the survival of 50–59-year old recipients, both in transplants from 18–49-year old donors (HR 2.24) and from ≥ 50-year old donors (HR 2.43). In 18–49-year old recipients, the survival of patients with pre-transplant diabetes was slightly inferior but the difference did not reach statistical significance, probably because of the lower number of diabetic patients in this age group. On the other hand, among ≥ 60-year old recipients, no difference in patient survival was observed between cases with or without pre-transplant diabetes. The explanation for this finding could be that most patients with more severe diabetes-related comorbidities could not survive long enough to reach the transplant because of increased mortality in the waitlist.

The association of longer time on dialysis and inferior patient survival has already been repeatedly reported in the literature (29–31). In the present study, time on dialysis ≥ 10 years conferred risk for patient survival in all donor-recipient ages combinations, except in transplants from younger donors into ≥ 60-year recipients. The explanation for this exception is probably related to the better quality of the younger kidneys and the implicit selection for healthier recipients during the prolonged time on dialysis.

In summary, the main results of our study were: (1) association of increased age of the donor with lower graft and patient survivals; (2) association of increased age of the recipient with higher graft survival and with lower patient survival; (3) no difference in graft survival between transplants in younger and older recipients when the donor was ≥ 60-year old; (4) impact of HLA mismatches on death-censored graft survival only in transplants from younger donors to younger recipients; (5) association of pre-transplant diabetes with lower patient survival only in 50–59-year old recipients; (6) association of time on dialysis ≥ 10 years with lower patient survival in transplants with all donor-recipient ages combinations, except in recipients with ≥ 60 years that received a kidney from a 18–49-year old donor.

This study has the limitation of being a single-center retrospective study in a relatively small cohort of 5,359 kidney transplants and with a limited number of factors that could be analyzed. In addition, some important factors could not be included, as the PRA (panel reactive antibody) because different methodologies for antibody determination have been used during the period covered by this study, socioeconomic variables, which are especially relevant in developing countries (43, 44) and cardiovascular disease, a very important risk factor for patient survival (42, 45).

In conclusion, this study has disclosed interesting interactions between age of the donor, age of the recipient and other factors that influence the survival of the graft and of the patient. Future multicentric studies, with large number of transplants, are warranted to further explore the impact of combinations of donor age with other risk factors to better understand and predict the impact of the age of the donor on kidney transplant outcomes.

## Data Availability Statement

The datasets generated for this study will not be made publicly available as the data are from a governmental state registry of transplantation data. Requests to access these datasets should be directed to Maria Gerbase-DeLima, gerbase@igen.org.br.

## Ethics Statement

Ethical review and approval was not required for the study on human participants in accordance with the local legislation and institutional requirements. Written informed consent for participation was not required for this study in accordance with the national legislation and the institutional requirements.

## Author Contributions

MG-D, KM, RM, FM, HT-S, and JM-P conceived and designed the study. MG-D and KM analyzed the data. MG-D and KM wrote the manuscript.

## Conflict of Interest

The authors declare that the research was conducted in the absence of any commercial or financial relationships that could be construed as a potential conflict of interest.
